# The role of autophagy in asparaginase-induced immune suppression of macrophages

**DOI:** 10.1038/cddis.2017.144

**Published:** 2017-03-30

**Authors:** Ping Song, Ziyu Wang, Xuyao Zhang, Jiajun Fan, Yubin Li, Qicheng Chen, Shaofei Wang, Peipei Liu, Jingyun Luan, Li Ye, Dianwen Ju

**Affiliations:** 1 Department of Microbiological and Biochemical Pharmacy & The Key Laboratory of Smart Drug Delivery, Ministry of Education, School of Pharmacy, Fudan University, 826 Zhangheng Road, Shanghai, 201203, P. R. China; 2 Department of Pharmacy, Ruijin Hospital Luwan Branch, Shanghai, 200020, P.R. China; 3 Department of Pharmacy, Huadong Hospital, Fudan University, Shanghai, 200040, P.R. China; 4 Department of Analytical Science, Sunshine Guojian Pharmaceutical (Shanghai) Co. Ltd, Shanghai, 201203, P.R. China

## Abstract

*Erwinia* asparaginase, a bacteria-derived enzyme drug, has been used in the treatment of various cancers, especially acute lymphoblastic leukemia (ALL). One of the most significant side effects associated with asparaginase administration is immune suppression, which limits its application in clinic. Macrophages are phagocytic immune cells and have a central role in inflammation and host defense. We reported here that asparaginase disturbed the function of macrophages including phagocytosis, proliferation, ROS and nitric oxide secretion, interleukin 6 (IL-6) and tumor necrosis factor *α* (TNF-*α*) secretion, and major histocompatibility complex II (MHC-II) molecule expression, thus induced immune suppression in interferon-*γ* and lipopolysaccharide-stimulated macrophages. We also observed that asparaginase inhibited autophagy in macrophages via activating Akt/mTOR and suppressing Erk1/2 signaling pathway as evidenced by less formation of autophagosomes, downregulation of autophagy-related protein LC3-II, and decreased number of autophagy-like vacuoles. Further study discovered that treatment with autophagy inhibitor 3-MA in place of asparaginase on activated macrophages could also downregulate phagocytosis, cytokine secretion, and MHC-II expression. Moreover, incubation with autophagy inducer trehalose restored the capacity of phagocytosis, IL-6 and TNF-*α* secretion, and MHC-II expression in macrophages. These results prove the important role of autophagy in the function of macrophages, and activation of autophagy can overcome asparaginase-induced immune suppression in macrophages.

Asparaginase, an enzyme drug derived from *Erwinia*, has been used for years in the treatment of acute lymphoblastic leukemia (ALL), myeloid leukemia, ovarian cancer, and T-cell lymphomas.^1, 2, 3, 4^ Asparaginase depletes asparagine in the blood by hydrolyzing the amino acid asparagine and glutamine to aspartic acid and glutamic acid, thus the malignant tumor cells that depend on extracellular asparagine fail to survive.^5^ Asparagine deprivation mediated by bacteria-derived asparaginase similarly causes suppression of cellular processes and pathways that involved in immune response, and consequently initiates side effects, which limits its application in clinic. Previous study has found that administration of *Escherichia coli* asparaginase to mice was rapidly immunosuppressive.^6^ The extranodal natural killer/T-cell lymphoma (ENKTL) patients receiving asparaginase-based chemotherapy who have more adverse clinical features showed increased serum levels of interleukin (IL)-10.^7^ IL-10 is a pleiotropic cytokine involved in the stimulation and suppression of immune response, and mounting evidence suggests that IL-10 can inhibit various immune functions including macrophage activation, nitric oxide (NO) synthesis, pro-inflammatory cytokine secretion, and antigen presentation.^8, 9, 10^ Asparaginase was reported to suppress T-cell blastogenesis, cytokine production, and proliferation and to downmodulate expression of the T-cell receptor, then limits T-cell responses.^11, 12^

Macrophages are an essential component of innate immunity and have a central role in inflammation and host defense.^13, 14^ Macrophages are extremely plastic cells, and may undergo classical M1 activation (stimulated by interferon (IFN)-*γ* and/or lipopolysaccharide; LPS) or alternative M2 activation (stimulated by IL-4/IL-13), which have distinct functional and phenotypical characteristics in response to various stimuli.^15, 16^ M1 macrophages exert pro-inflammatory and anti-tumor function characterized by high expression of pro-inflammatory cytokines, chemokines, reactive nitrogen intermediates, and reactive oxygen intermediates. By contrast, M2 macrophages have anti-inflammatory and pro-wound healing activities.^17, 18, 19^ Despite impressive advances in the understanding of its immune suppression, whether asparaginase influences the function of macrophages is rarely addressed. Because of the important role of macrophages in the immune system, we employed IL-4 to activate macrophages to generate M2 macrophages, and found that asparaginase did not influence the IL-10 secretion in M2 macrophages. We speculated that asparaginase might mainly affect the function of M1 macrophages.^20^

To verify this hypothesis, we used IFN-*γ* and LPS to stimulate macrophages to generate M1 macrophages. We found that asparaginase not only inhibited phagocytosis and proliferation of macrophage, but also downregulated autophagy in macrophages. Autophagy is a highly conserved cellular process in eukaryotes to maintain cellular homeostasis by supporting cell survival and regulating inflammation.^21^ Autophagy degrades unnecessary or dysfunctional intracellular components, such as abnormal proteins, old organelles, and pathogens, and has been widely studied in various immune cells, including T cell, B cell, macrophages, dendritic cells (DCs), and neutrophils.^22, 23, 24, 25^ Recent studies suggest an important role of autophagy in regulating the immune response of macrophages. Macrophage autophagy regulates inflammation and insulin sensitivity.^26, 27^ In obese mice, high-fat diet conditions (HFD)-induced inflammatory conditions downregulate autophagy in macrophages, which promote the production of reactive oxygen species (ROS) and pro-inflammatory cytokines, further aggravating inflammation.^28^ Macrophages also participate in the process of atherosclerosis, and macrophage autophagy contributes to the inhibition of foam cell formation by reducing oxidized low density lipoprotein ingestion and inflammasome activation, and increasing efferocytosis and cholesterol efflux in macrophages.^29, 30^ All of these results prompt us to explore the role of autophagy in asparaginase-induced immune suppression of macrophages.

In this study, we demonstrate that *Erwinia* asparaginase induces obvious immune suppression as well as autophagy downregulation in IFN-*γ* and LPS-stimulated macrophages. Further study elucidates that inhibition of autophagy restrains the function of macrophages including cytokine secretion, major histocompatibility complex II (MHC-II) expression and phagocytosis, and activating autophagy by autophagy inducer trehalose (Tre) can overcome asparaginase-induced immune suppression in macrophages. Our results advance knowledge of the influence of asparaginase on M1 macrophages, and provide an insight into the role of autophagy in asparaginase-induced immune suppression.

## Results

### Asparaginase inhibits phagocytosis and proliferation in M1 macrophages

**Figure 1:**
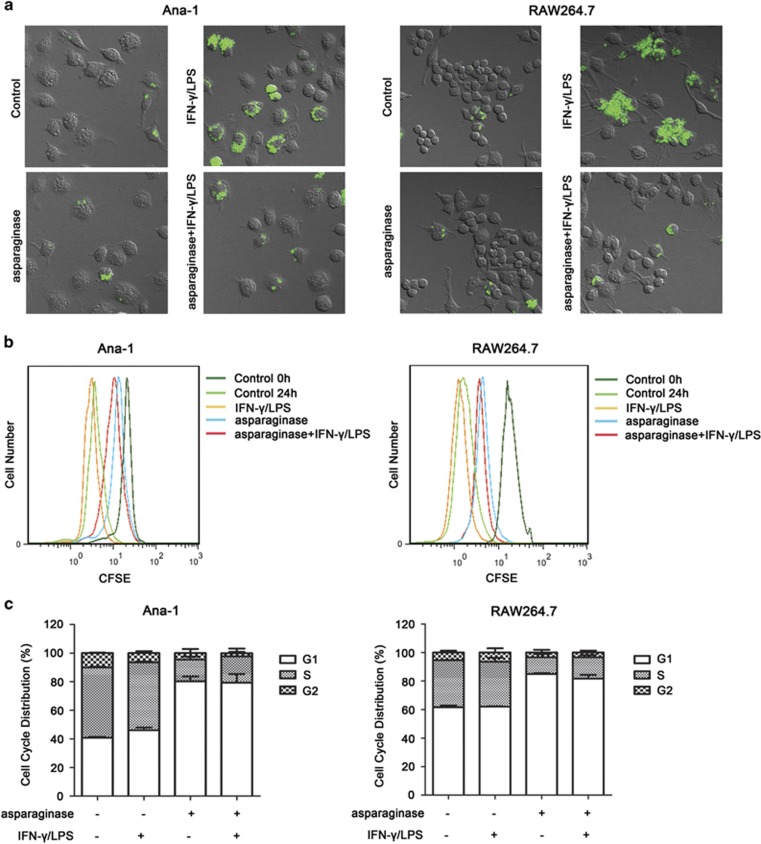
Phagocytosis and proliferation are inhibited by asparaginase in Ana-1 and RAW264.7 cells. (**a**) Ana-1 and RAW264.7 cells were treated with 300 IU/ml IFN-*γ* and 200 ng/ml LPS, either alone or in combination with 0.1 IU/ml asparaginase for 24 h. The cells were incubated with zymosan particles for another 2 h, and analyzed by confocal fluorescent microscopy. (**b**) Ana-1 and RAW264.7 cells were labeled with CFSE, then incubated with 300 IU/ml IFN-*γ* and 200 ng/ml LPS, either alone or in combination with 0.1 IU/ml asparaginase for 24 h. The fluorescence intensity was analyzed by flow cytometry. Cells without treatment were used as control. (**c**) Ana-1 and RAW264.7 cells were treated with 300 IU/ml IFN-*γ* and 200 ng/ml LPS, in the presence or absence of 0.1 IU/ml asparaginase for 24 h, cell cycle distribution were analyzed by flow cytometry. The percentage of cells in different phases was presented in bar graphs

First, the effect of asparaginase on macrophages phagocytic function was determined in activated Ana-1 and RAW264.7 cells. Phagocytosis is a basic cellular function of macrophages having an important role in innate immunity.^31^ As shown in Figure 1a and Supplementary Figure 1, Ana-1 and RAW264.7 cells stimulated by IFN-*γ* and LPS for 24 h showed significantly increased phagocytic activity measured as zymosan-Alexa Fluor 488 particles internalization (green fluorescence). However, the macrophage phagocytic function was significantly impaired when treated with asparaginase, indicating the phagocytosis was inhibited by asparaginase in activated Ana-1 and RAW264.7 cells.

Second, we labeled macrophages with carboxyfluorescein succinimidyl ester (CFSE) and investigated the effect of asparaginase on proliferation of Ana-1 and RAW264.7 cells.^32^ As shown in Figure 1b, at 0 h in control, Ana-1 and RAW264.7 cells showed a potent fluorescence intensity (right side), and at 24 h in both control and IFN-*γ*/LPS-treated cells the fluorescence intensity attenuated owing to cell division (left side). After treating with asparaginase, the curve shifted from left to the right as compared with control and IFN-*γ*/LPS-treated cells, indicating that asparaginase inhibited proliferation of macrophages.

Finally, we investigated the effect of asparaginase on cell cycle distribution in Ana-1 and RAW264.7 cells. Cell cycle is a repeating series of events that take place in a cell leading to its division and DNA replication to produce two daughter cells. As shown in Figure 1c, upon asparaginase treatment, Ana-1 and RAW264.7 cells at G1 phase increased with reduced cells at S phase when compared with IFN-*γ* and LPS-stimulated macrophages, indicating asparaginase could induce G1 arrest to decelerate the cell cycle, and prevent the cells from entering the S phase and proliferating.

These results demonstrate that asparaginase inhibits phagocytosis and proliferation in M1 macrophages, and support our hypothesis that asparaginase might influence the function of M1 macrophages.

### Asparaginase inhibits ROS and NO generation in M1 macrophages

**Figure 2:**
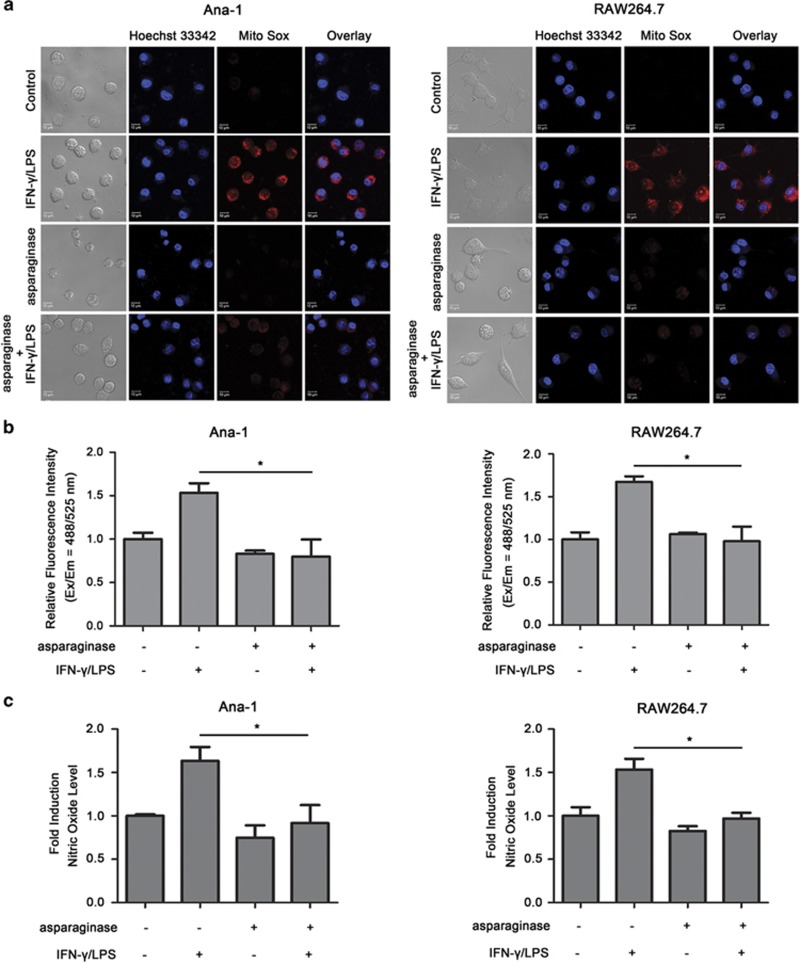
ROS and NO generation are inhibited by asparaginase in Ana-1 and RAW264.7 cells. (**a**–**c**) Ana-1 and RAW264.7 cells were treated with 300 IU/ml IFN-*γ* and 200 ng/ml LPS, either alone or in combination with 0.1 IU/ml asparaginase for 24 h. (**a**) Cells were stained with Mito Sox red dye (ROS) and examined by confocal fluorescent microscopy. (**b**) Intracellular ROS levels in macrophages were determined by relative fluorescence intensity. (**c**) NO production was assessed by Griess reagent, microplate reader was used to determine the OD value. Results were represented as mean±S.D. (*P<0.05)

Activated macrophages generally have high rate of free radical secretion (superoxide, NO), which helps to destroy foreign material. We determined the level of ROS and NO in asparaginase-treated Ana-1 and RAW264.7 cells to investigate whether asparaginase influences free radical secretion in macrophages. First of all, Ana-1 and RAW264.7 cells stimulated with IFN-*γ* and LPS either alone or in combination with asparaginase for 24 h were stained by Hoechst 33342 (nucleus) and Mito sox (ROS), and followed by measurement of fluorescence of Ana-1 and RAW264.7 cells using confocal microscopy. Figure 2a and Supplementary Figure 2 showed that activated Ana-1 and RAW264.7 cells exhibited potent intensity of red fluorescence, which indicates high level of ROS, whereas, when treated with asparaginase, the fluorescence intensity reduced obviously. To further determine the ROS level in asparaginase-treated Ana-1 and RAW264.7 cells, we used 20,70-dichlorofluorescein diacetate (DCFH-DA) assay. As expected, we indeed found a significant reduction of ROS level, expressed by relative fluorescence intensity, in macrophages incubated with asparaginase for 24 h when compared with activated macrophages (Figure 2b). Next, Griess reagent was used to detect the NO level in Ana-1 and RAW264.7 cells. As shown in Figure 2c, IFN-*γ* and LPS stimulation significantly increased the production of NO by ~1.6-fold when compared with the untreated cells. However, the IFN-*γ* and LPS-induced NO production reduced in the Ana-1 and RAW264.7 cells when treated with asparaginase (*P*<0.05), indicating asparaginase restrained NO production in IFN-*γ* and LPS-stimulated macrophages.

These observations strongly suggest that asparaginase inhibits ROS and NO generation in M1 macrophages.

### The cytokine secretion and MHC-II expression are inhibited by asparaginase in M1 macrophages

**Figure 3:**
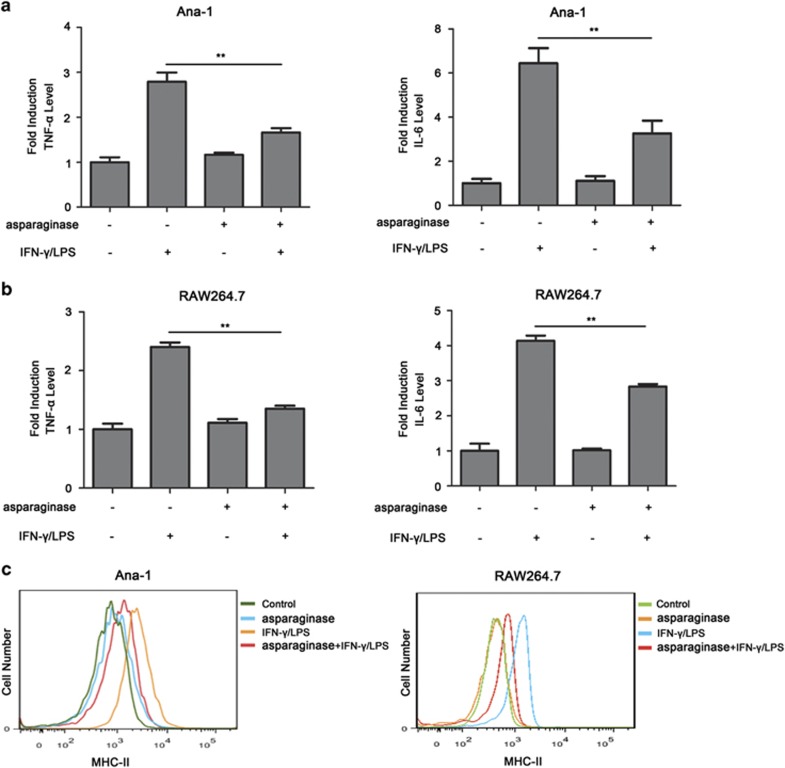
Cytokine production and MHC-II expression are inhibited by asparaginase in Ana-1 and RAW264.7 cells. (**a**–**c**) Ana-1 or RAW264.7 cells were treated with 300 IU/ml IFN-*γ* and 200 ng/ml LPS, in the presence or absence of 0.1 IU/ml asparaginase for 24 h. (**a**) The content of TNF-*α* and IL-6 in the supernatants of Ana-1 cells was measured by ELISA. (**b**) The levels of TNF-*α* and IL-6 in the culture medium of RAW264.7 cells was measured by ELISA. (**c**) MHC-II expression of Ana-1 and RAW264.7 cells was detected by flow cytometer. Results were represented as mean±S.D. (**P<0.01)

The pro-inflammatory cytokine production is one of the most important functions of activated macrophages by inflammatory stimulation. The effect of asparaginase on two pro-inflammatory cytokines, tumor necrosis factor *α* (TNF-*α*) and IL-6, was investigated in IFN-*γ* and LPS-stimulated macrophages. As depicted in Figure 3a, in Ana-1 macrophages, incubation with IFN-*γ* and LPS alone for 24 h significantly increased the secretion of TNF-*α* and IL-6 by 2.8 and 6.4-fold, respectively, compared with non-treated cells as measured by enzyme-linked immunosorbent assay (ELISA) kit. However, the secretion of TNF-*α* and IL-6 decreased when treated with asparaginase (*P*<0.01). In RAW264.7 macrophages, stimulation with IFN-*γ* and LPS for 24 h significantly increased the production of TNF-*α* and IL-6 by 2.5 and 4.1-fold, respectively, and treatment with 0.1 IU/ml asparaginase in macrophages decreased the secretion of TNF-*α* and IL-6 (*P*<0.01) (Figure 3b). Similarly, in peritoneal macrophages, incubation with IFN-*γ* and LPS alone for 24 h significantly increased the secretion of TNF-*α* and IL-6 by 2.7 and 5.5-fold, respectively, and treatment with 0.1 IU/ml asparaginase decreased the secretion of TNF-*α* and IL-6 (Supplementary Figure 3) (*P*<0.01).

MHC-II is constitutively expressed in the surface of DCs, monocytes and macrophages. As MHC-II presents antigens to CD4^+^ T cells, it is the main regulator of the immune response.^33, 34^ We cannot help asking whether asparaginase inhibits surface expression of MHC-II in macrophages. As shown in Figure 3c, stimulation of Ana-1 and RAW264.7 cells with IFN-*γ* and LPS resulted in increased surface expression of MHC-II. However, when treated with asparaginase, MHC-II surface expression in activated Ana-1 and RAW264.7 cells significantly decreased, indicating asparaginase inhibited intracellular presentation of antigenic peptides to T cells via MHC-II.

Together, these results demonstrate that asparaginase inhibits cytokine secretion and MHC-II expression in M1 macrophages.

### Autophagy is downregulated in M1 macrophages during asparaginase treatment

**Figure 4:**
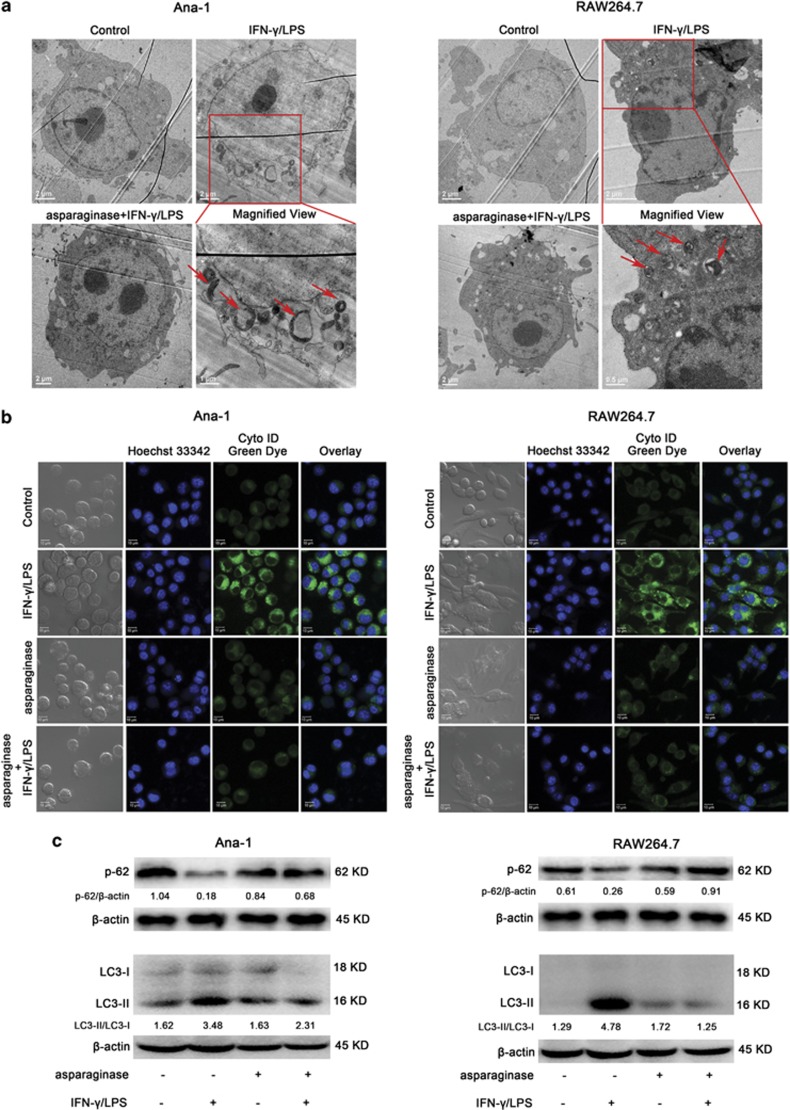
Autophagy is downregulated by asparaginase in Ana-1 and RAW264.7 cells. (**a**–**c**) Ana-1 and RAW264.7 cells were treated with 300 IU/ml IFN-*γ* and 200 ng/ml LPS, either alone or in combination with 0.1 IU/ml asparaginase for 24 h. (**a**) TEM was employed to detect the autophagosomes (‘red arrows': autophagosomes) in Ana-1 and RAW264.7 cells. (**b**) Macrophages were stained with Cyto-ID Green autophagy dye and examined by confocal fluorescent microscopy. (**c**) Autophagy-associate protein LC3-I/II and p62 were detected by western blot analysis. Densitometric values were quantified using the ImageJ software, and the data represented mean of three independent experiments

Recent studies have demonstrated that autophagy has a beneficial role in the function of macrophages as a major regulator of immune responses such as clearance of intracellular pathogens, antigen presentation and excessive production of inflammatory cytokines.^35, 36^ To examine whether asparaginase influences autophagic response in activated Ana-1 and RAW264.7 cells, several well-established methods were performed to detect autophagosomes formation. First of all, we examined the intracellular morphologic change of Ana-1 and RAW264.7 cells using transmission electron microscopy (TEM). Figure 4a showed increased accumulation of double-membrane-enclosed autophagosomes in both Ana-1 and RAW264.7 cells after IFN-*γ* and LPS stimulation for 24 h, whereas the number of autophagic vacuoles decreased when treated with asparaginase. Next, we used a Cyto-ID Green dye autophagy detection kit to detect autophagic vacuoles with fluorescence microscopy. Ana-1 and RAW264.7 cells, stimulated with IFN-*γ* and LPS for 24 h, displayed more green fluorescence than that in the non-treated cells. In contrast, when incubated with 0.1 IU/ml asparaginase, the activated Ana-1 and RAW264.7 cells showed limited specific green fluorescence (Figure 4b and Supplementary Figure 4). Finally, we examined the conversion of microtubule-associated protein LC3 (known as autophagy-related protein) and Sequestosome 1 (SQSTM1/p62) (a substrate of autophagy), to assess autophagy levels in Ana-1 and RAW264.7 cells through western blot analysis. Autophagosome formation involves the conversion of LC3 from the cytosolic LC3-I to the autophagosome-associated LC3-II form and decrease of p62 levels.^21^ As shown in Figure 4c, IFN-*γ* and LPS-stimulated macrophages exhibited an obvious conversion of endogenous LC3-I to LC3-II and decrease in protein levels of p62, whereas combined treatment with 0.1 IU/ml asparaginase downregulated the LC3-II conversion and increased p62 levels in activated macrophages.

Together, these results indicate that asparaginase downregulates the formation of autophagosomes in M1 macrophages.

### Asparaginase downregulates autophagy by activating Akt/mTOR and suppressing Erk1/2 signaling pathway in M1 macrophages

**Figure 5:**
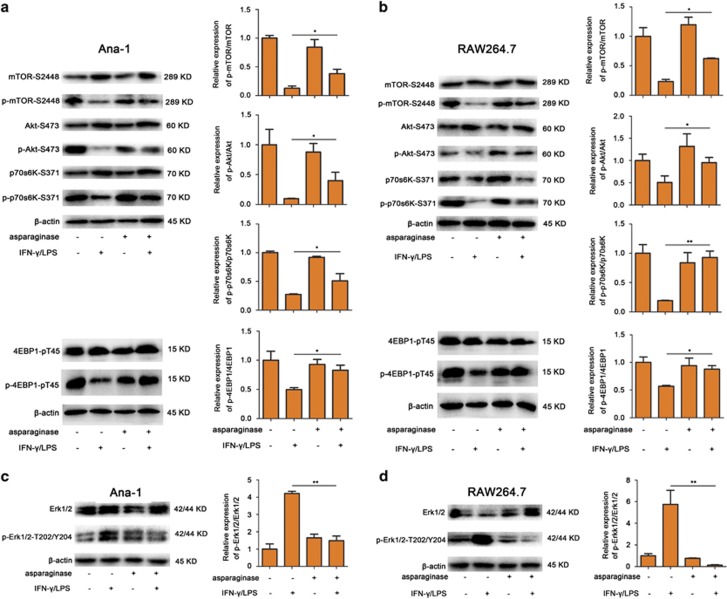
Asparaginase downregulates autophagy by activating Akt/mTOR and inhibiting Erk1/2 signaling pathway. (**a**–**d**) Ana-1 and RAW264.7 cells were treated with 300 IU/ml IFN-*γ* and 200 ng/ml LPS, either alone or in combination with 0.1 IU/ml asparaginase for 24 h. (**a**) The expression level of mTOR, p-mTOR, Akt, p-Akt, p70s6K, p-p70s6K, 4EBP1, and p-4EBP1 in Ana-1 cells were analyzed by western blot. Quantification of p-mTOR/mTOR, p-Akt/Akt, p-p70s6K/p70s6K, and p-4EBP1/4EBP1 ratios in different groups were presented in bar graphs. (**b**) The expression of mTOR, p-mTOR, Akt, p-Akt, p70s6K, p-p70s6K, 4EBP1, and p-4EBP1 in RAW264.7 cells were analyzed by western blot. Quantification of p-mTOR/mTOR, p-Akt/Akt, p-p70s6K/p70s6K, and p-4EBP1/4EBP1 ratios in different groups were presented in bar graphs. (**c**) Western blot was performed to analyze the protein Erk1/2 and p-Erk1/2 in Ana-1 cells. Quantification of p-Erk1/2/Erk1/2 ratios in different groups were presented in bar graphs. (**d**) Western blot was performed to analyze the protein Erk1/2 and p-Erk1/2 in RAW264.7 cells. Quantification of p-Erk1/2/Erk1/2 ratios in different groups were presented in bar graphs. Densitometric values were quantified using the ImageJ software and normalized to control. The values of control were set to 1. Results were represented as mean±S.D. (*P<0.05, **P<0.01)

We further explored the underlying mechanism of asparaginase-induced autophagy downregulation in Ana-1 and RAW264.7 cells. The mammalian target of rapamycin (mTOR), an evolutionarily conserved serine/threonine kinase, serves as not only a key positive regulator of protein synthesis and cell growth but also a major negative regulator of autophagy in eukaryotic cells.^37^ MTOR can be phosphorylated by upstream protein phosphorylated-Akt to form p-mTOR-S2448, which inhibits autophagy. Moreover, mTOR downregulates autophagy by mediating protein translation and cell growth through the phosphorylation of two key protein targets, eukaryotic initiation factor 4E-binding protein 1 (4EBP1) and polypeptide 1 ribosomal protein S6 kinase-1 (p70s6K).^38^ Our experiments discovered that asparaginase-treated Ana-1 and RAW264.7 cells expressed high level of protein p-mTOR-S2448 when compared with IFN-*γ* and LPS-activated macrophages. Besides, asparaginase efficiently increased the phosphorylation of Akt (upstream protein of mTOR), p70s6K and 4EBP1 (two downstream substrates of mTOR) (Figures 5a and b). Moreover, after treating with asparaginase, the ratios of p-mTOR/mTOR, p-Akt/Akt, p-p70s6K/ p70s6K, and p-4EBP1/4EBP1 increased as compared with IFN-*γ*/LPS-treated macrophages (*P*<0.05, *P*<0.01), indicating mTOR activity was activated in macrophages after exposure to asparaginase.

Because extracellular signal-regulated kinase (Erk1/2) has been shown to regulate expression of autophagy and lysosomal genes,^38^ we then analyzed whether asparaginase modulated the expression of Erk1/2 pathway-related protein in Ana-1 and RAW264.7 cells. Figures 5c and d showed that the expression of p-Erk1/2 in the macrophages stimulated with IFN-*γ* and LPS was significantly higher than that in non-stimulated cells. The addition of asparaginase alone to macrophages had little influence on p-Erk1/2 expression. However, it significantly weakened IFN-*γ* and LPS-induced p-Erk1/2 expression. Furthermore, after treating with asparaginase, the ratios of p-Erk1/2/Erk1/2 decreased as compared with IFN-*γ* and LPS-activated macrophages (*P*<0.01), indicating Erk1/2 activity was suppressed in macrophages after exposure to asparaginase.

Together, these experiments suggest that asparaginase downregulates autophagy in M1 macrophages by activating Akt/mTOR and suppressing Erk1/2 signaling pathway.

### Autophagy has an important role in asparaginase-induced immune suppression in M1 macrophages

**Figure 6:**
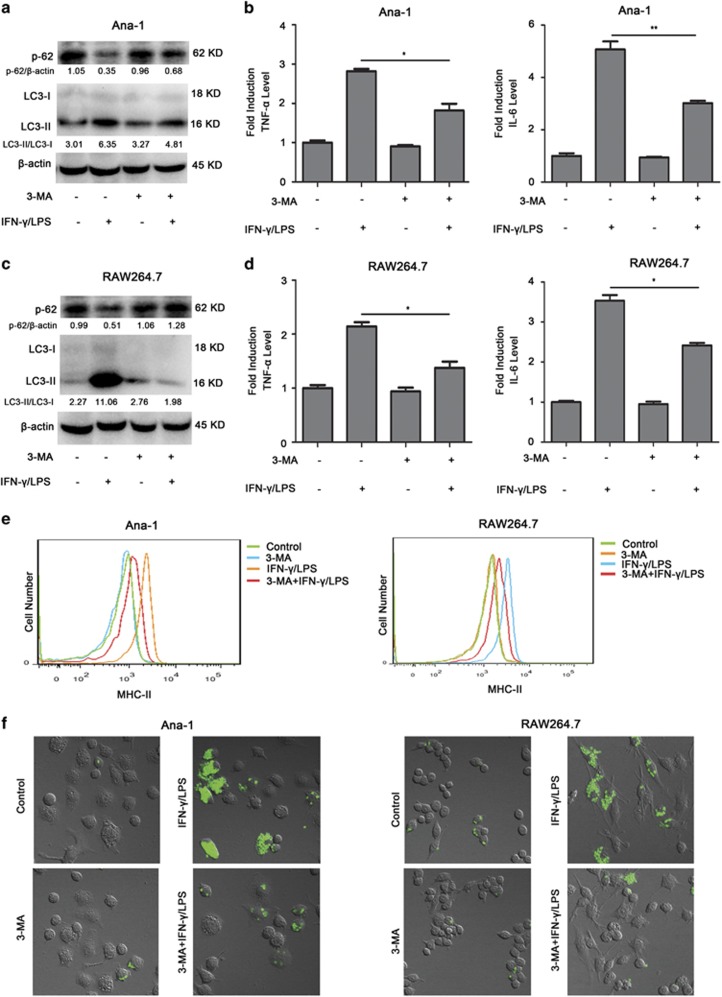
Inhibiting autophagy induces immune suppression in Ana-1 and RAW264.7 cells. (**a**–**f**) Ana-1 and RAW264.7 cells were treated with 300 IU/ml IFN-*γ* and 200 ng/ml LPS, in the presence or absence of 2 mM 3-MA for 24 h. (**a**) Autophagy-associated protein LC3-I/II and p62 were detected by western blot analysis in Ana-1 cells. Densitometric values were quantified using the ImageJ software, and the data represented mean of three independent experiments. (**b**) The content of TNF-*α* and IL-6 in the supernatants of Ana-1 cells were measured by ELISA. (**c**) Autophagy-associated protein LC3-I/II and p62 were detected by western blot analysis in RAW264.7 cells. Densitometric values were quantified using the ImageJ software, and the data represented mean of three independent experiments. (**d**) The level of TNF-*α* and IL-6 in the culture medium of RAW264.7 cells were measured by ELISA. (**e**) The expression of MHC-II in Ana-1 and RAW264.7 cells were detected by flow cytometer. (**f**) The cells were incubated with zymosan particles for another 2 h, and analyzed by confocal fluorescent microscopy. Results were represented as mean±S.D. (*P<0.05, **P<0.01)

To reveal the role of autophagy in the weakening effects of asparaginase on IFN-*γ* and LPS-induced IL-6, TNF-*α* secretion, MHC-II expression, and phagocytosis in Ana-1 and RAW264.7 cells, the autophagy inhibitor 3-methyladenine (3-MA) was used in place of asparaginase. 3-MA is a specific inhibitor of PI3K, which inhibits autophagosomes accumulation and inhibits the conversion of LC3-I to LC3-II. Western blot analysis indicated that IFN-*γ* and LPS-induced autophagy was successfully inhibited by 2 mM 3-MA (Figures 6a and c). As shown in Figures 6b, d and e, and Supplementary Figure 5, at 24 h after 3-MA treatment, the secretion of TNF-*α* and IL-6, and the expression of MHC-II in IFN-*γ* and LPS-stimulated macrophages markedly decreased. We next examined the effect of 3-MA on phagocytosis of macrophages. As shown in Figure 6f and Supplementary Figure 6, inhibition of autophagy by 3-MA suppressed the phagocytosis of activated macrophages, indicating autophagy had an important role in the function of macrophages.

**Figure 7:**
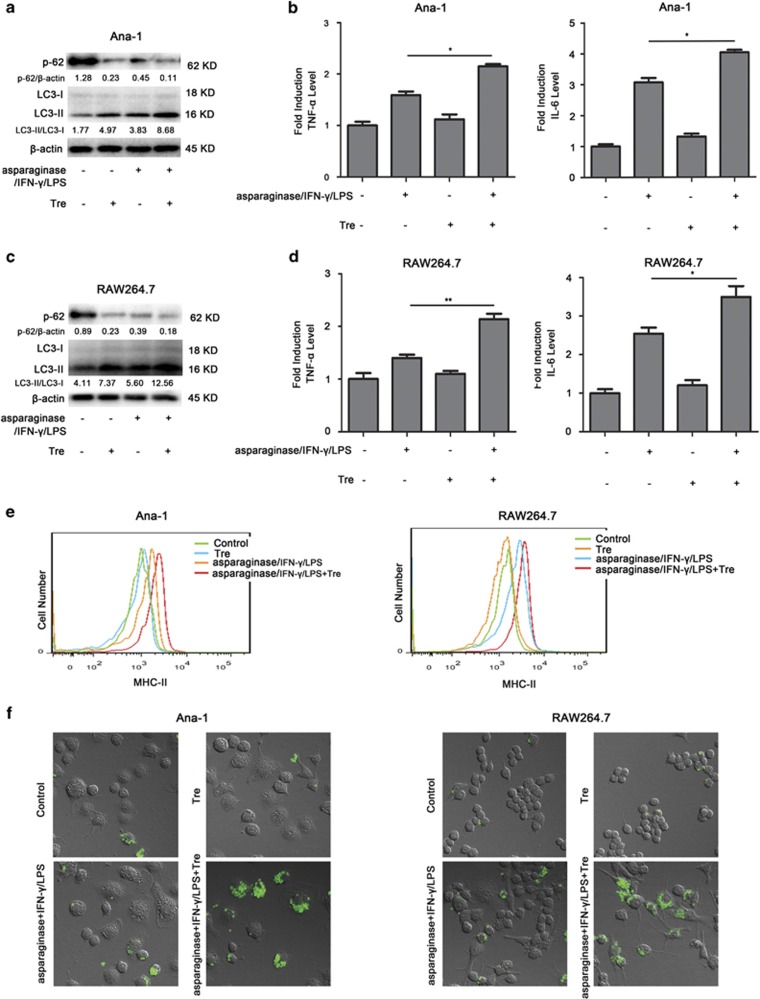
Activating autophagy overcomes asparaginase-induced immune suppression in Ana-1 and RAW264.7 cells. (**a**–**f**) Ana-1 and RAW264.7 cells were treated with 300 IU/ml IFN-*γ*, 200 ng/ml LPS, and 0.1 IU/ml asparaginase, either alone or in combination with 25 *μ*M Tre for 24 h. (**a**) Autophagy-associated protein LC3-I/II and p62 were detected by western blot analysis in Ana-1 cells. Densitometric values were quantified using the ImageJ software, and the data represented mean of three independent experiments. (**b**) The content of TNF-*α* and IL-6 in the supernatants of Ana-1 cells were measured by ELISA. (**c**) Autophagy-associated protein LC3-I/II and p62 were detected by western blot analysis in RAW264.7 cells. Densitometric values were quantified using the ImageJ software, and the data represented mean of three independent experiments. (**d**) The level of TNF-*α* and IL-6 in the culture medium of RAW264.7 cells were measured by ELISA. (**e**) MHC-II expression of Ana-1 and RAW264.7 cells were detected by flow cytometer. (**f**) The cells were incubated with zymosan particles for another 2h, and analyzed by confocal fluorescent microscopy. Results were represented as mean±S.D. (*P<0.05, **P<0.01)

As asparaginase simultaneously inhibited immune function and autophagic response in activated Ana-1 and RAW264.7 cells, and it has been reported that autophagy had a beneficial role in the function of macrophages, we want to know whether activation of autophagy could overcome asparaginase-induced immune suppression in Ana-1 and RAW264.7 cells. In our following experiments, Tre (an autophagy inducer) was combined with asparaginase to treat IFN-*γ* and LPS-stimulated Ana-1 and RAW264.7 cells. Western blot analysis showed that Tre could upregulate the conversion of LC3-II and decrease the levels of p62 compared with asparaginase-treated activated macrophages (Figures 7a and c). Subsequently, we found that in activated Ana-1 and RAW264.7, as well as in peritoneal macrophages, Tre significantly increased the secretion of pro-inflammatory mediators TNF-*α* and IL-6 compared with asparaginase-treated macrophages (*P*<0.05, *P*<0.01) (Figures 7b and d and Supplementary Figure 7). In addition, we observed Tre could restore the expression of MHC-II and phagocytosis of Ana-1 and RAW264.7 cells, which inhibited by asparaginase (Figures 7e and f and Supplementary Figure 8).

All of these results suggest that autophagy has an important role in the function of M1 macrophages. Activation of autophagy can overcome asparaginase-induced immune suppression in M1 macrophages.

## Discussion

*Erwinia* asparaginase is an FDA-approved enzyme drug for treatment of select cancers. Asparaginase administration results in rapid and complete deamination of the amino acid asparagine and, to a lesser extent, glutamine, leading to depletion of asparagine in the plasma.^39^ Despite its application as an essential drug used in all treatment protocols for ALL, asparaginase is associated with a unique set of side effect that limits its clinical outcomes. When asparaginase therapy was introduced in the treatment of cancer it became obvious that this bacteria-derived enzyme had other effects, one of the most significant is immunosuppression, which is regarded to be in association with its glutaminase activity.^6^ Immune cells require substantial amounts of glutamine to sustain its proliferation and functions, whereas all the asparaginases possess some degree of glutaminase activity, which hydrolyzes the amino acid glutamine.^40^

Previous studies have reported the inhibition effect of asparaginase on blastogenesis and proliferation of T cell, and expression of T-cell receptor.^11, 12^ However, its effects on macrophages functions, especially the underlying molecular mechanism, are still not fully understood. Macrophages are phagocytic immune cells that reside in most tissues and thus constitute the first line of host defense, and participate in all kinds of the progress of inflammatory diseases, such as atherosclerosis, bacterial and virus infection, obesity-induced insulin resistance, and diabetes.^28, 30, 41, 42^ They are metabolically active cells characterized by high levels of phagocytosis, cytokine secretion, free radical secretion, and antigen presentation-related molecular expression. All of these processes constitute the overall function of the macrophage destroying foreign material via exposure to free radicals, antigen presentation to T-lymphocytes via MHC-II molecules and activation of lymphocyte subpopulations by cytokine.^17, 43^

To discover the effect of asparaginase on macrophage function, we first adopted IL-4 to activate macrophages to generate M2 macrophages, and found that asparaginase treatment had no obvious effect on the secretion of IL-10 in M2 macrophages, which suggested that asparaginase did not influence the function of M2 macrophages (Supplementary Figure 9). We then used IFN-*γ* and LPS to stimulate macrophages to generate M1 macrophages, and examined the changes of phagocytosis, proliferation, free radical secretion, pro-inflammatory cytokine secretion, and MHC-II molecule expression in macrophages upon asparaginase treatment. We observed that asparaginase significantly impaired macrophage phagocytic function and induced G1 arrest to prevent the cells from proliferating. Through confocal, DCFH-DA assay and Griess reagent assay, we also found that ROS and NO generation in activated Ana-1 and RAW264.7 cells decreased, indicating that asparaginase disturbed free radical secretion function of macrophage. Moreover, the results of ELISA and flow cytometer manifested that the secretion of two pro-inflammatory cytokine, TNF-*α* and IL-6, and the expression of antigen presentation molecule MHC-II were all downregulated. These results are in agreement with previous reports that enzyme-induced glutamine depletion restrained MHC-II expression, antigen presentation and phagocytosis, and LPS-stimulated TNF-*α* and IL-6 production in murine macrophage was also dependent upon the availability of extracellular glutamine.^6, 40^

Our further experiment proved that asparaginase treatment had no obvious effect on the number of macrophages, indicating the immune suppression effect of asparaginase was not by reducing the number of macrophages (Supplementary Figure 10). In recent years, a growing body of evidence demonstrates the important role of autophagy in innate immunity, inflammatory responses, and adaptive immunity.^25, 44^ These findings inspire us to reveal whether asparaginase affects autophagic response of macrophages, furthermore, whether asparaginase induces macrophage immune suppression by influencing autophagy. In our experiments, we indeed observed that asparaginase inhibited autophagic response in activated Ana-1 and RAW264.7 cells as evidenced by the less formation of autophagosomes through TEM analysis, downregulation of autophagy-related protein LC3-II through western blot analysis, and decreased number of autophagy-like vacuoles through confocal microscopy analysis.

We also discovered the underlying molecular mechanisms of asparaginase-induced autophagy inhibition. The Akt/mTOR and Erk1/2 signaling pathway is two of the major pathways regulating autophagy in eukaryotic cells.^45^ MTOR acts as a major negative regulator of autophagy, whereas Erk1/2 positively regulates expression of autophagy genes.^46, 47^ In our study, western blot analysis showed that mTOR activity was activated in macrophages by asparaginase as evidenced by increased phosphorylation of mTOR, upstream protein Akt, and two downstream substrates p70s6K and 4EBP1, meanwhile Erk1/2 signaling pathway was suppressed evidenced by decreased p-Erk1/2 expression. We came to a conclusion that asparaginase inhibited autophagy in macrophages by activating Akt/mTOR and suppressing Erk1/2 signaling pathway.

Based on above experiments, we demonstrate that asparaginase not only suppresses immune function but also inhibits autophagic response in activated macrophages. In consideration of the importance and complexity of autophagy in immune system,^48, 49^ it is necessary to elucidate the role of autophagy in asparaginase-induced immune suppression of macrophages. We first used autophagy inhibitor 3-MA to replace asparaginase, and examined its effect on the function of macrophages including phagocytosis, IL-6 and TNF-*α* secretion and MHC-II expression. As a result, the same effect was achieved by 3-MA. Subsequently, an autophagy inducer Tre was adopted in combination with asparaginase to treat activated macrophages. It was very inspiring that overcoming asparaginase-induced autophagy inhibition by Tre restored the capacity of phagocytosis, IL-6 and TNF-*α* secretion and MHC-II expression in macrophages. These results suggest a key role of autophagy in asparaginase-induced immune suppression in macrophages.

In conclusion, our present study demonstrates that asparaginase disturbes the function of macrophages including phagocytosis, proliferation, free radical secretion, cytokine secretion, and MHC-II molecule expression, thus induces immune suppression. Moreover, asparaginase inhibits autophagy in macrophages by activating Akt/mTOR and suppressing Erk1/2 signaling pathway. Further investigations suggest that asparaginase induces macrophage immune suppression through influencing autophagy, and combined treatment with autophagy inducer can reverse asparaginase-induced immune suppression. Our research highlights the important role of autophagy in asparaginase-induced immune suppression in macrophages.

## Materials and methods

### Materials

Asparaginase (derived from *Erwinia*) was purchased from Baiyunshan Mingxing Pharmaceutical Co., Ltd. (Guangzhou, Guangdong Province, China). The autophagy inhibitor 3-MA was obtained from EMD Chemicals, Inc. (San Diego, CA, USA). The autophagy inducer Tre was purchased from Sigma-Aldrich (St. Louis, MO, USA). IFN-*γ* was obtained from Cyagen Biosciences Inc. (Guangzhou, Guangdong Province, China). LPS was obtained from Sigma-Aldrich. The antibodies including anti-*β*-actin, anti-LC3B, anti-p62, anti-mTOR (Ser2448), anti-phospho-mTOR (Ser2448), anti-Akt (Ser473), anti-phospho-Akt (Ser473), anti-p70S6 Kinase (pS371), anti-p70S6 Kinase Phospho (pS371), anti-4EBP1-pT45, anti-phospho-4EBP1-pT45, anti-phospho-p44/42 MAPK (Erk1/2) (Thr202/Tyr204), and anti-p44/42 MAPK (Erk1/2) were purchased from Cell Signaling Technology (Danvers, MA, USA). The secondary antibodies horseradish peroxidases (HRP)-conjugated goat anti-mouse and anti-rabbit immunoglobulin G were purchased from MR Biotech (Shanghai, China).

### Cell culture

C57BL/6 male mice were purchased from Shanghai Super-B&K Laboratory Animal Corp., Ltd. (Shanghai, China). 5% starch broth was used to stimulated inflammatory peritoneal macrophages in mice for 3 days, then peritoneal macrophages were isolated and cultured in RPMI-1640 containing 10% heat-inactivated fetal bovine serum (FBS) (Invitrogen, San Diego, CA, USA). Murine Ana-1 and RAW264.7 cells were purchased from Cell Bank of Chinese Academy of Sciences, Shanghai Branch (Shanghai, China). The cells were cultured in RPMI-1640 or Dulbecco's modified eagle's medium (DMEM) (Invitrogen) containing 10% FBS, 100 U/ml of penicillin, and 100 *μ*g/ml of streptomycin at 37 °C in a humidified atmosphere of 5% CO_2_ incubator.

### Phagocytosis assay

Ana-1 and RAW264.7 cells were plated in glass bottom cell culture dishes (NEST Biotechnology, Jiangsu, China) at a density of 1 × 10^5^/ml. After treatments, cells were incubated with zymosan A BioParticles Alexa Fluor 488 (Thermo, Z23373) for 2 h following the manufacturer's instructions. Then cells were washed with phosphate-buffered saline (PBS) to remove non-phagocytosis fluorescent particles. The samples were observed by confocal microscope (Carl Zeiss LSM710, Carl Zeiss, Oberkochen, Germany). The percentage of zymosan-positive macrophages was presented in bar charts.

### Western blot analysis

To determine the levels of autophagy-related protein in Ana-1 and RAW264.7 cells, total protein contents were analyzed by western blot as described previously.^2^ The protein bands were incubated with specialized antibodies, and identified with HRP-conjugated secondary antibodies. Detection was performed with enhanced chemiluminescence (ECL) detection kit (Pierce, Rockford, IL, USA). Intensities in the resulting bands were quantified by ChemiDoc software (Bio-Rad, CA, USA).

### Cell cycle analysis

The distribution in the cell cycle phases was determined by FACS Calibur flow cytometer (Becton-Dickinson, Fullerton, CA, USA) analysis. Ana-1 and RAW264.7 cells were treated with 300 IU/ml IFN-*γ* and 200 ng/ml LPS, either alone or in combination with 0.1 IU/ml asparaginase for 24 h. Then, the cells were harvested and fixed in 70% ethanol at the temperature of −20 °C for overnight. The cells were washed with PBS to remove fixative and stained with PI and RNaseA at 4 °C for 30 min. The samples were analyzed by FACS Calibur flow cytometer.

### Cell proliferation analysis

The cell proliferation was determined by CFDA SE cell proliferation and tracking kit (Beyotime Institute of Biotechnology, Haimen, China). Ana-1 and RAW264.7 cells were harvested and labeled with CFSE according to the manufacturer instructions. Labeled cells were seeded in six-well plates at a density of 2 × 10^5^ cells/well. Then, the cells were incubated with 300 IU/ml IFN-*γ* and 200 ng/ml LPS, either alone or in combination with 0.1 IU/ml asparaginase for 24 h. Proliferation was analyzed using FACS Calibur flow cytometer and the resulting data was analyzed by Flow Jo 10.0 program.

### Transmission electron microscopy analysis

TEM assays were performed as described in our previous study.^50^ Ana-1 and RAW264.7 cells were incubated with 300 IU/ml IFN-*γ* and 200 ng/ml LPS, either alone or in combination with 0.1 IU/ml asparaginase for 24 h, then harvested and fixed with ice-cold glutaraldehyde. Samples were detected with a JEM 1410 transmission electron microscope (JEOL, Inc., MA, USA) at 80 kV.

### Confocal microscopy

Ana-1 and RAW264.7 cells were plated in glass bottom cell culture dishes at a density of 1 × 10^5^/ml. After 24 h of incubation, cells were treated with 300 IU/ml IFN-*γ* and 200 ng/ml LPS, either alone or in combination with 0.1 IU/ml asparaginase for another 24 h. Then, cells were stained with Cyto-ID Green dye and Hoechst 33342 at 37 °C for 30 min following the manufacturer's instruction. All the procedures were done in the dark place and the samples were observed by an inverted confocal microscope.

### Measurement of NO production

Ana-1 and RAW264.7 cells were treated with 300 IU/ml IFN-*γ* and 200 ng/ml LPS, either alone or in combination with 0.1 IU/ml asparaginase for 24 h. The cell supernatants were collected and assayed for NO production using Griess reagent (Beyotime Institute of Biotechnology). In brief, the samples were mixed with an equal volume of Griess reagent I and Griess reagent II. The optical density (OD) was measured at an absorbance wavelength of 540 nm. Sodium nitrite (0–100 *μ*M) was used as a standard to assess nitrite concentrations.

### ROS measurement

Reactive oxygen species assay kit (Beyotime Biotechnology, Haimen, China) was used to detect intracellular ROS generation in macrophages. Ana-1 and RAW264.7 cells were seeded into 96-well black plates and treated with 300 IU/ml IFN-*γ* and 200 ng/ml LPS, either alone or in combination with 0.1 IU/ml asparaginase. After 24 h incubation, 10 *μ*M of the fluorescent probe DCFH-DA was added to each well, and the cells were incubated at 37 °C for 20 min. Subsequently, cells were washed twice with serum-free medium. A microplate reader was used to determine the OD value (excitation: 488 nm, emission: 525 nm).

### Cytokine analysis

Macrophages were treated with 300 IU/ml IFN-*γ* and 200 ng/ml LPS, either alone or in combination with 0.1 IU/ml asparaginase. After 24 h of incubation, the cell supernatants were collected and assayed for cytokine production using ELISA Kit (Boatman Biotech, Shanghai, China). The assay was performed according to the manufacturer's instructions.

### MHC-II expression analysis

Ana-1 and RAW264.7 cells were treated with 300 IU/ml IFN-*γ* and 200 ng/ml LPS, either alone or in combination with 0.1 IU/ml asparaginase for 24 h. The cells were harvested and incubated with an antibody against FCGR2/CD32 and FCGR3/CD16 (FcR*γ* blocker, BD Biosciences, San Diego, CA, USA) for 5 min, followed by a PE-conjugated I-A/I-E antibody (BD Biosciences) for additional 30 min at 37 °C. Then, the cells were analyzed immediately by using a FACS Calibur flow cytometer (Becton-Dickinson).

### Microscopy and photography

About 1 × 10^4^ Ana-1 and RAW264.7 cells were seeded into 96-well plates. After incubation for 24 h, cells were examined by using an inverted microscope (Nikon, Tokyo, Japan) equipped with a model digital camera.

### Statistical analysis

All data were presented as mean±S.D.s. Statistical analysis was performed using Student's *t*-test. *, **, and *** indicated *P*<0.05, *P*<0.01, and *P*<0.001, respectively.
