# Supplementary material for: The role of autophagy in asparaginase-induced immune suppression of macrophages
Source: Cell Death Dis. 2017 Mar 30;8(3):e2721–. doi: 10.1038/cddis.2017.144 (PMC5386542; doi:10.1038/cddis.2017.144)
Supplement: Supplementary Information [file cddis2017144x1.docx]

**The role of autophagy in asparaginase-induced immune suppression of macrophages**

Ping Song^1,2,*^, Ziyu Wang^1,3,*^, Xuyao Zhang^1,*^, Jiajun Fan^1^, Yubin Li^1^, Qicheng Chen^1^, Shaofei Wang^1^, Peipei Liu^1,4^, Jingyun Luan^1^, Li Ye^1^, Dianwen Ju^1^

**Supplementary Figures**

**
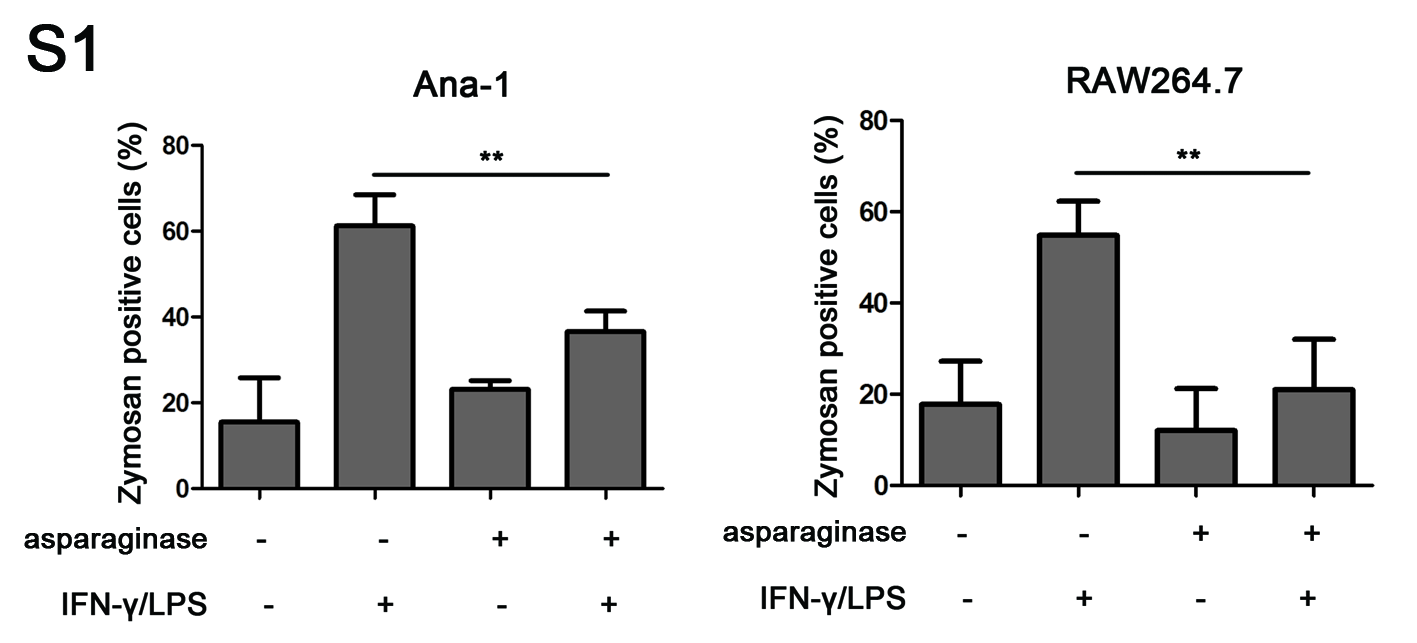
**

**Supplementary Figure 1. Phagocytosis is inhibited by asparaginase in Ana-1 and RAW264.7 cells.** Ana-1 and RAW264.7 cells were treated with 300 IU/mL IFN-γ and 200 ng/mL LPS, either alone or in combination with 0.1 IU/mL asparaginase for 24 h. The cells were incubated with zymosan particles for another 2 h, and analyzed by confocal fluorescent microscopy, The percentage of zymosan positive macrophages was presented in bar charts. Results were represented as mean ± SD (***P* < 0.01).


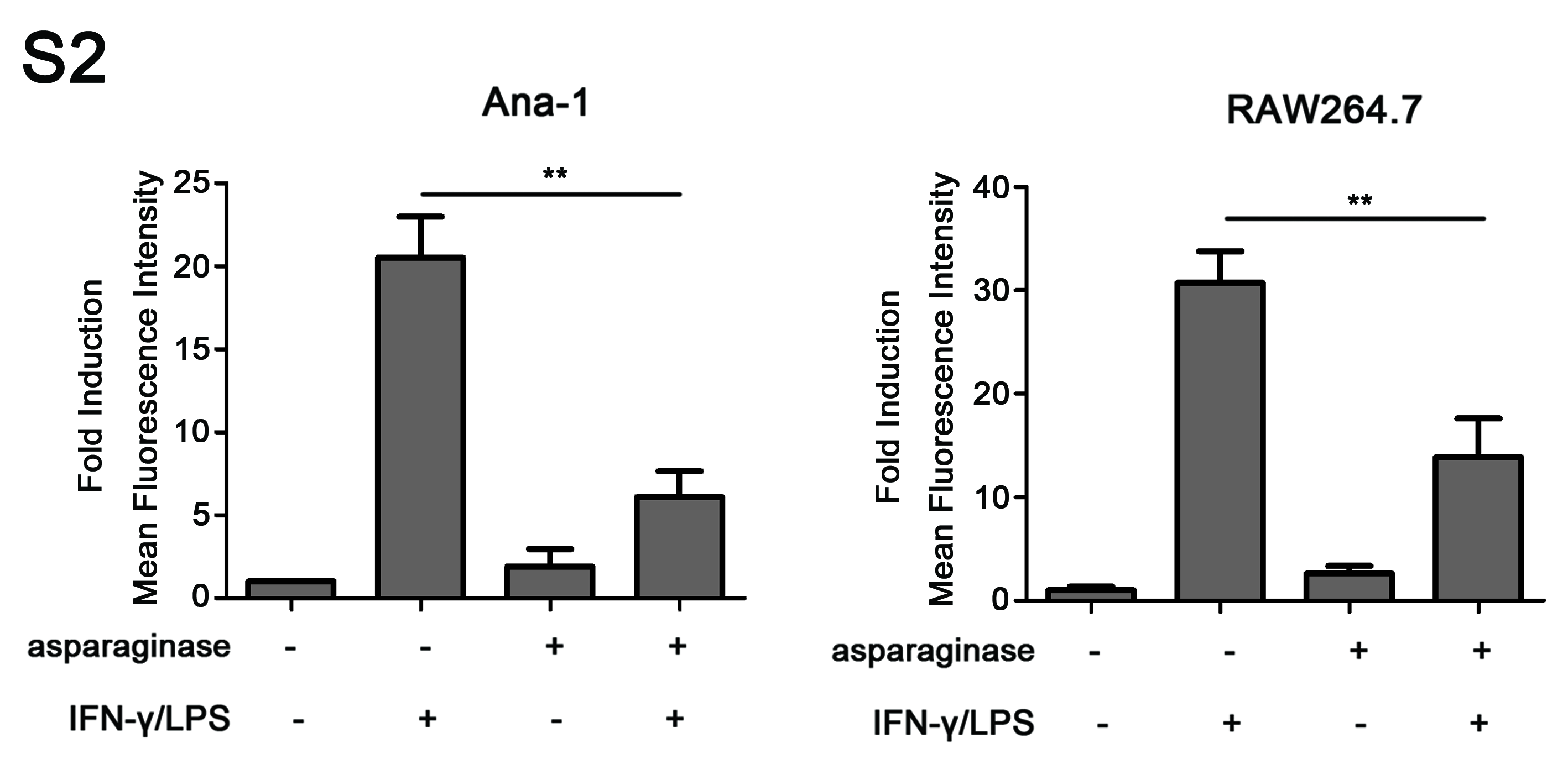


**Supplementary Figure 2. ROS generation is inhibited by asparaginase in Ana-1 and RAW264.7 cells.** Ana-1 and RAW264.7 cells were treated with 300 IU/mL IFN-γ and 200 ng/mL LPS, either alone or in combination with 0.1 IU/mL asparaginase for 24 h. Cells were stained with Mito Sox red dye (ROS) and examined by confocal fluorescent microscopy. Red dots in cells were represented as mean ± SD (***P* < 0.01).


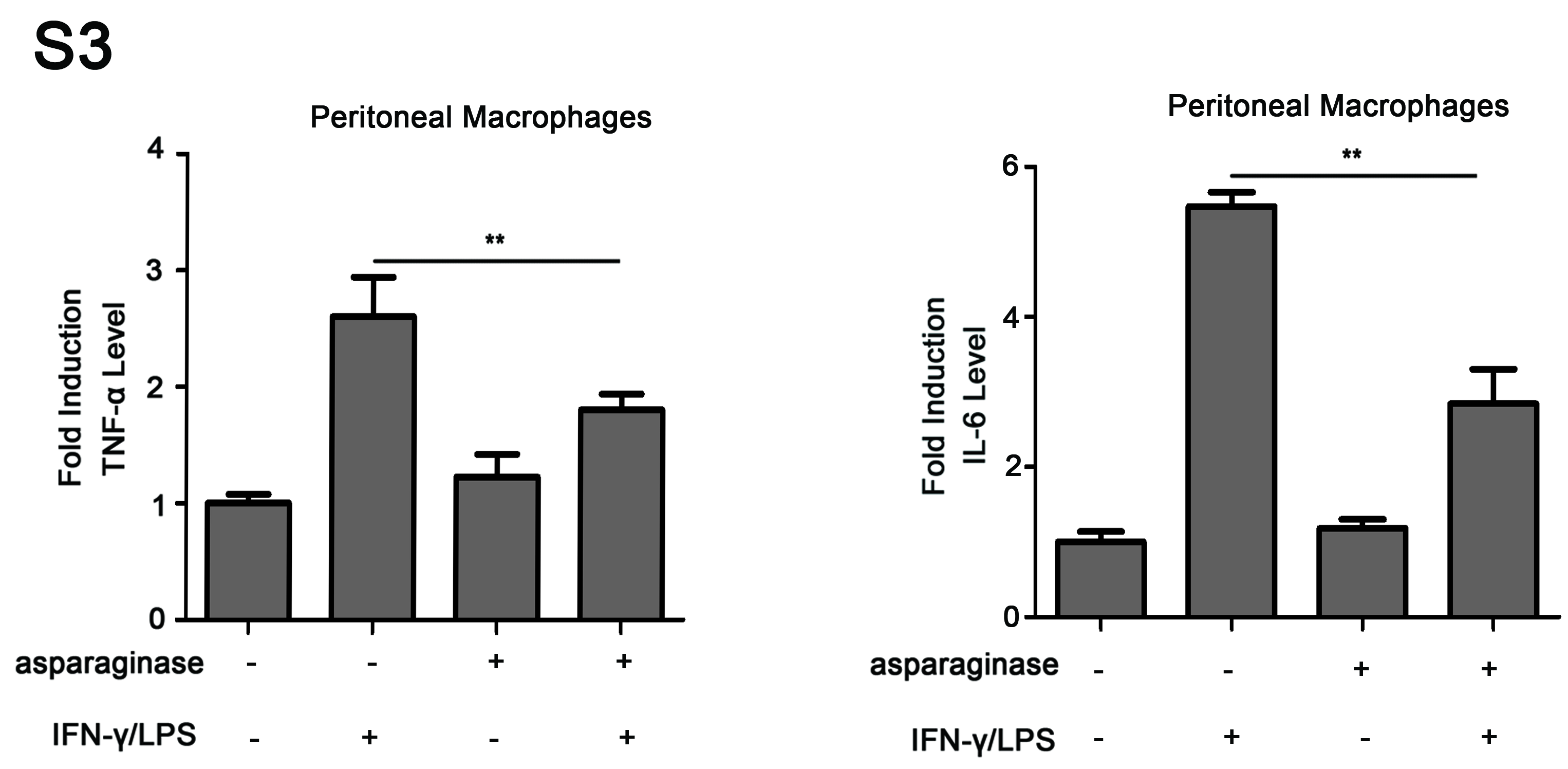


**Supplementary Figure 3. [Cytokine](file:///F:\\宋平文章资料\\宋平投稿用\\CDD\\javascript:void(0);) production is inhibited by asparaginase in [peritoneal](D:/%E8%BD%AF%E4%BB%B6/Dict/6.3.69.8341/resultui/frame/javascript:void(0);) [macrophage](D:/%E8%BD%AF%E4%BB%B6/Dict/6.3.69.8341/resultui/frame/javascript:void(0);)s.** [Peritoneal](D:/%E8%BD%AF%E4%BB%B6/Dict/6.3.69.8341/resultui/frame/javascript:void(0);) [macrophage](D:/%E8%BD%AF%E4%BB%B6/Dict/6.3.69.8341/resultui/frame/javascript:void(0);)s were treated with 300 IU/mL IFN-γ and 200 ng/mL LPS, in the presence or absence of 0.1 IU/mL asparaginase for 24 h. The content of TNF-α and IL-6 in the supernatants of [peritoneal](D:/%E8%BD%AF%E4%BB%B6/Dict/6.3.69.8341/resultui/frame/javascript:void(0);) [macrophage](D:/%E8%BD%AF%E4%BB%B6/Dict/6.3.69.8341/resultui/frame/javascript:void(0);)s was measured by ELISA. Results were represented as mean ± SD (***P* < 0.01).


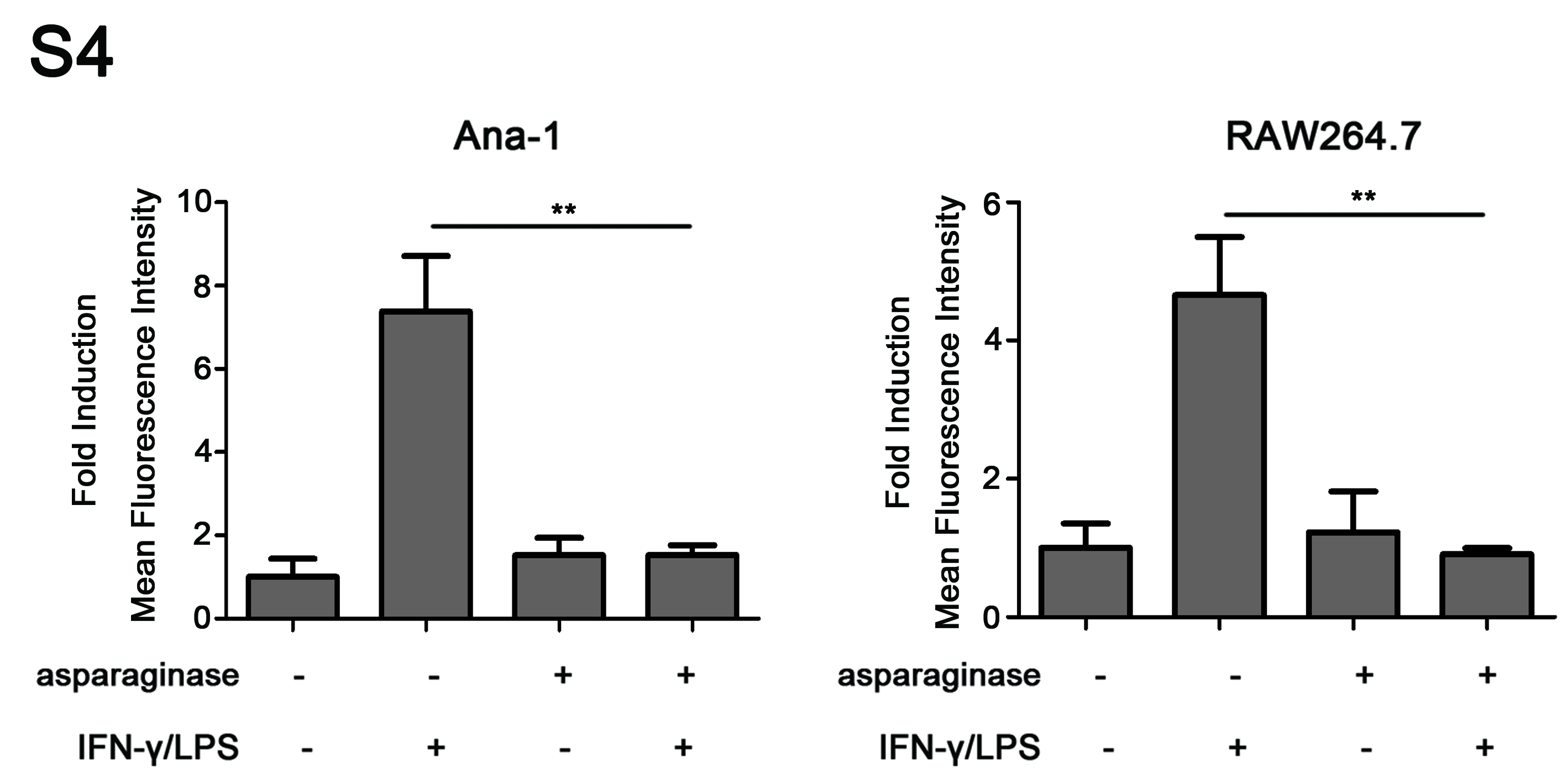


**Supplementary Figure 4. Autophagy is downregulated by asparaginase** **in Ana-1 and RAW264.7 cells.** Ana-1 and RAW264.7 cells were treated with 300 IU/mL IFN-γ and 200 ng/mL LPS, either alone or in combination with 0.1 IU/mL asparaginase for 24 h. Macrophages were stained with Cyto-ID Green autophagy dye and examined by confocal fluorescent microscopy. Green dots in cells were represented as mean ± SD (***P* < 0.01).


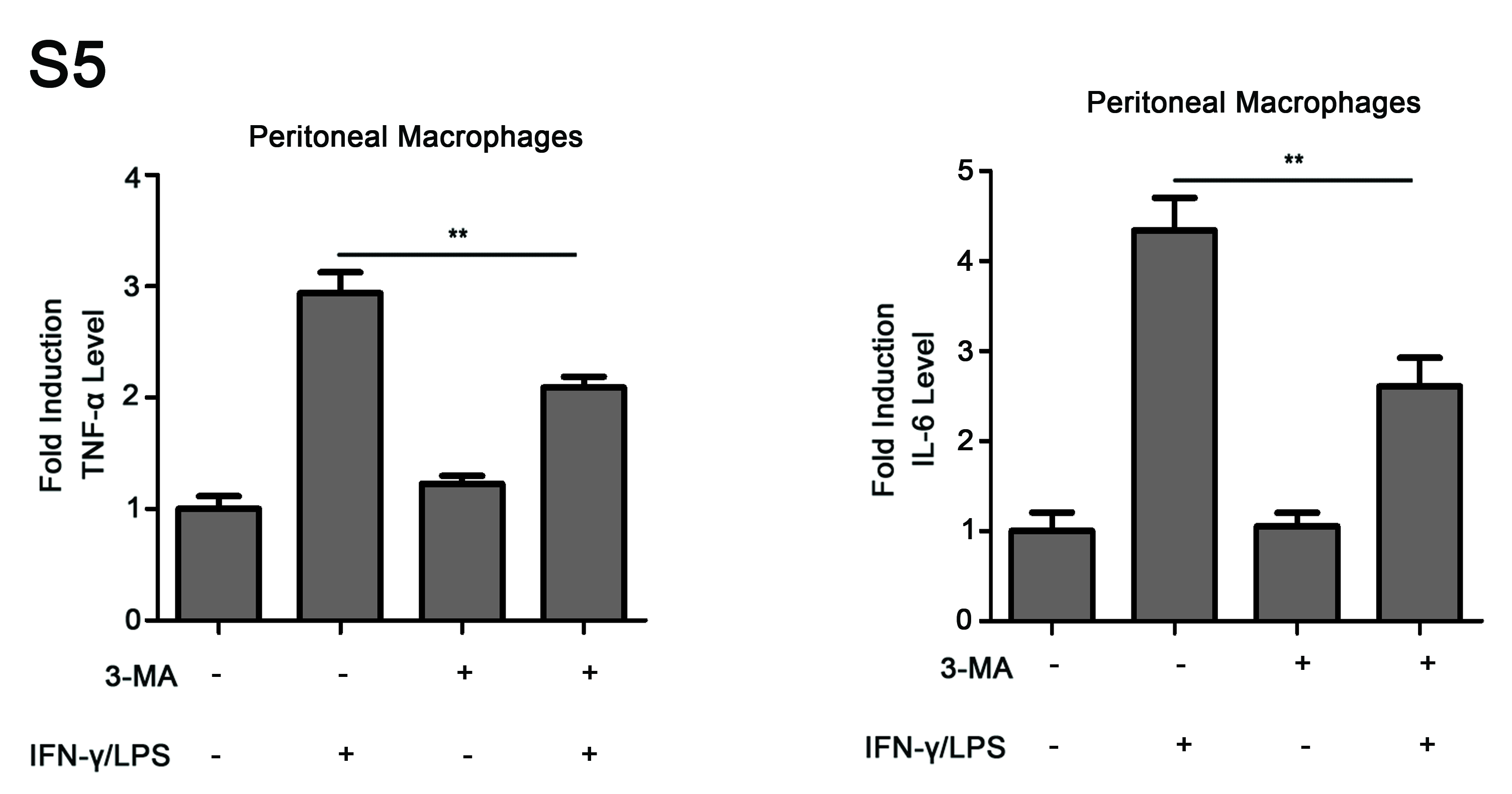


**Supplementary Figure 5. Suppressing autophagy inhibits [cytokine](file:///F:\\宋平文章资料\\宋平投稿用\\CDD\\javascript:void(0);) secretion in [peritoneal](D:/%E8%BD%AF%E4%BB%B6/Dict/6.3.69.8341/resultui/frame/javascript:void(0);) [macrophage](D:/%E8%BD%AF%E4%BB%B6/Dict/6.3.69.8341/resultui/frame/javascript:void(0);)s.** [Peritoneal](D:/%E8%BD%AF%E4%BB%B6/Dict/6.3.69.8341/resultui/frame/javascript:void(0);) [macrophage](D:/%E8%BD%AF%E4%BB%B6/Dict/6.3.69.8341/resultui/frame/javascript:void(0);)s were treated with 300 IU/mL IFN-γ and 200 ng/mL LPS, in the presence or absence of 2 mM 3-MA for 24 h. The content of TNF-α and IL-6 in the supernatants of [peritoneal](D:/%E8%BD%AF%E4%BB%B6/Dict/6.3.69.8341/resultui/frame/javascript:void(0);) [macrophage](D:/%E8%BD%AF%E4%BB%B6/Dict/6.3.69.8341/resultui/frame/javascript:void(0);)s were measured by ELISA. Results were represented as mean ± SD (***P* < 0.01).


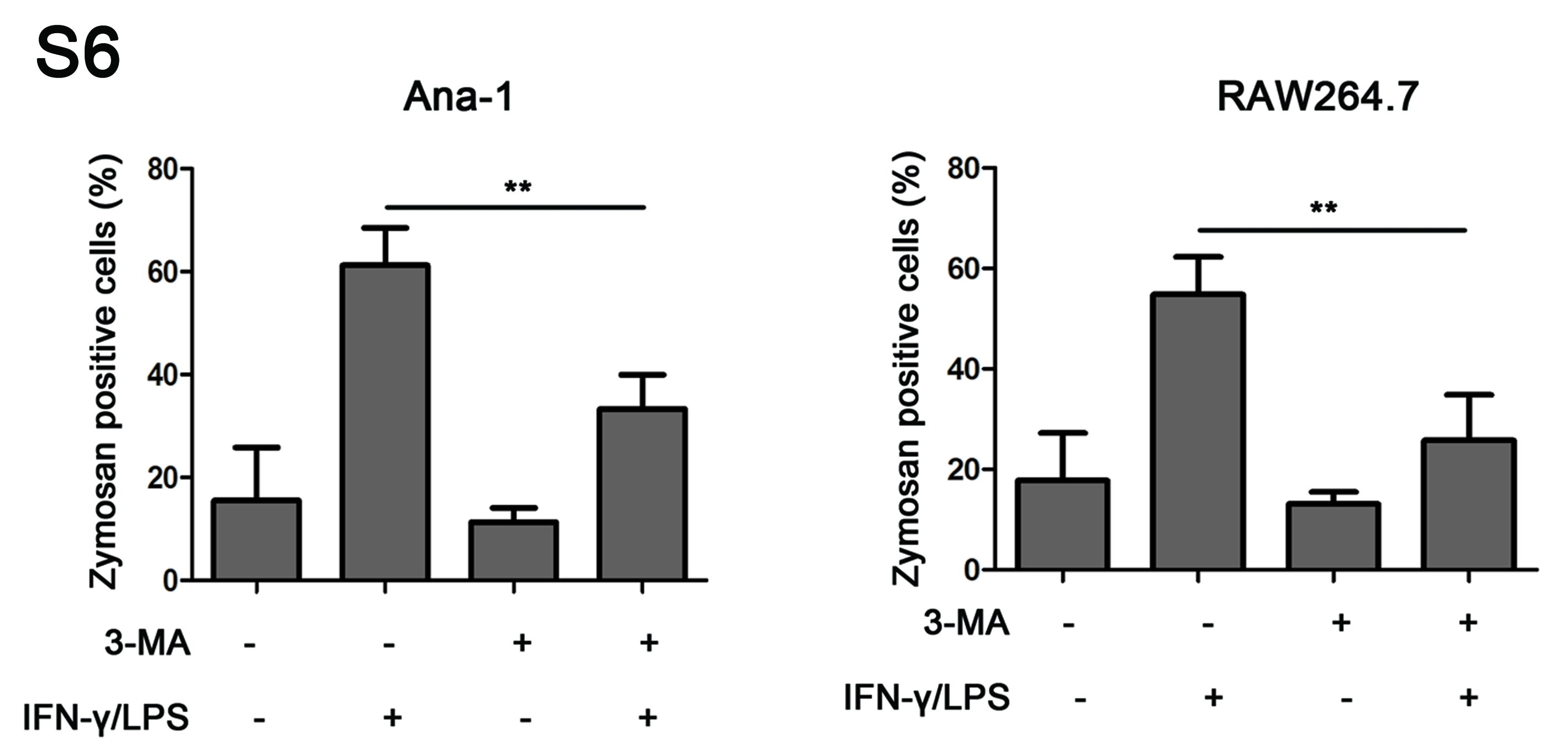


**Supplementary Figure 6. Suppressing autophagy inhibits phagocytosis in Ana-1 and RAW264.7 cells.** Ana-1 and RAW264.7 cells were treated with 300 IU/mL IFN-γ and 200 ng/mL LPS, in the presence or absence of 2 mM 3-MA for 24 h. The cells were incubated with zymosan particles for another 2 h, and analyzed by confocal fluorescent microscopy. The percentage of zymosan positive macrophages was presented in bar charts. Results were represented as mean ± SD (***P* < 0.01).


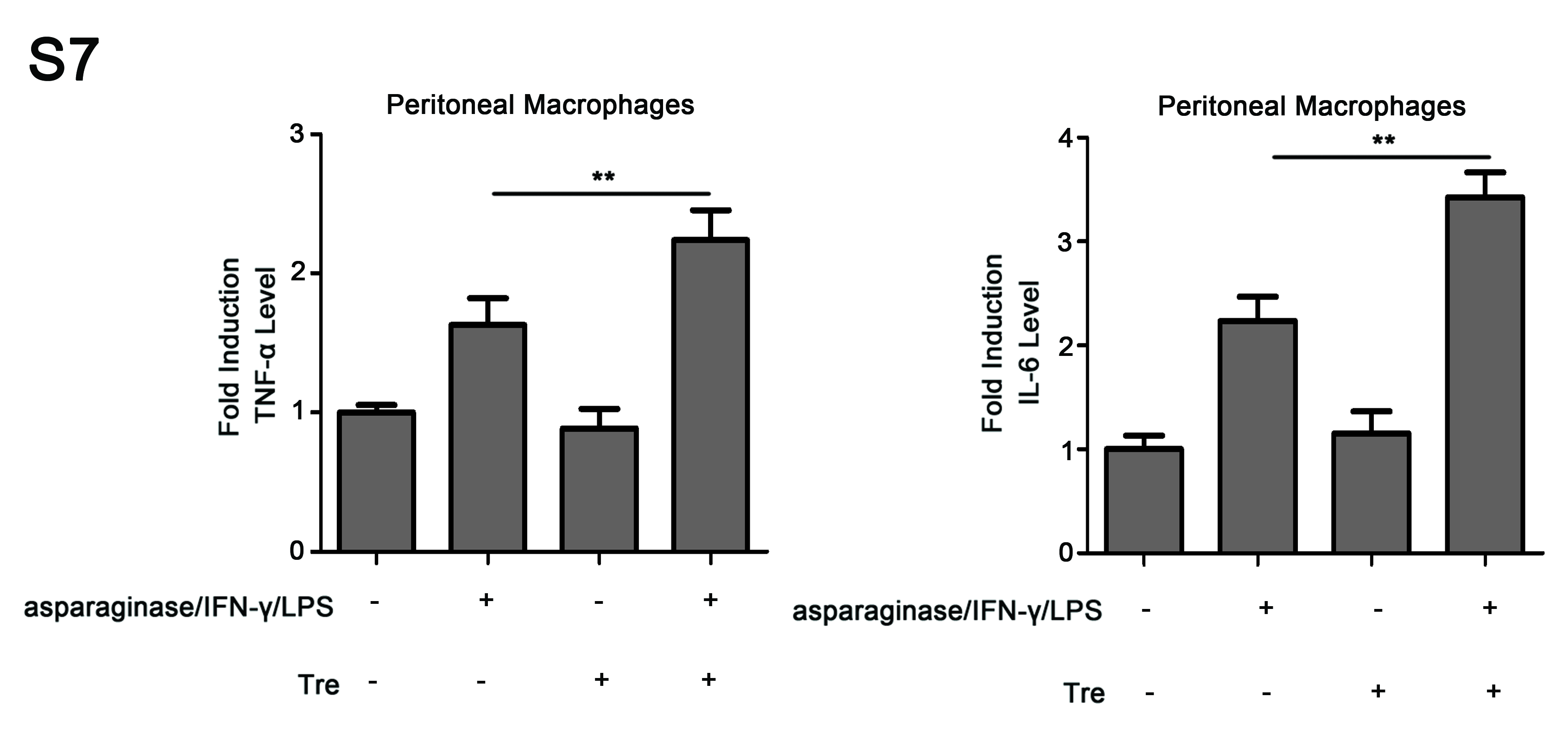


**Supplementary Figure 7. Activating autophagy overcomes asparaginase-induced immune suppression in [peritoneal](D:/%E8%BD%AF%E4%BB%B6/Dict/6.3.69.8341/resultui/frame/javascript:void(0);) [macrophage](D:/%E8%BD%AF%E4%BB%B6/Dict/6.3.69.8341/resultui/frame/javascript:void(0);)s.** [Peritoneal](D:/%E8%BD%AF%E4%BB%B6/Dict/6.3.69.8341/resultui/frame/javascript:void(0);) [macrophage](D:/%E8%BD%AF%E4%BB%B6/Dict/6.3.69.8341/resultui/frame/javascript:void(0);)s were treated with 300 IU/mL IFN-γ, 200 ng/mL LPS and 0.1 IU/mL asparaginase, either alone or in combination with 25 µM Tre for 24 h. The content of TNF-α and IL-6 in the supernatants of [peritoneal](D:/%E8%BD%AF%E4%BB%B6/Dict/6.3.69.8341/resultui/frame/javascript:void(0);) [macrophage](D:/%E8%BD%AF%E4%BB%B6/Dict/6.3.69.8341/resultui/frame/javascript:void(0);)s were measured by ELISA. Results were represented as mean ± SD (***P* < 0.01).


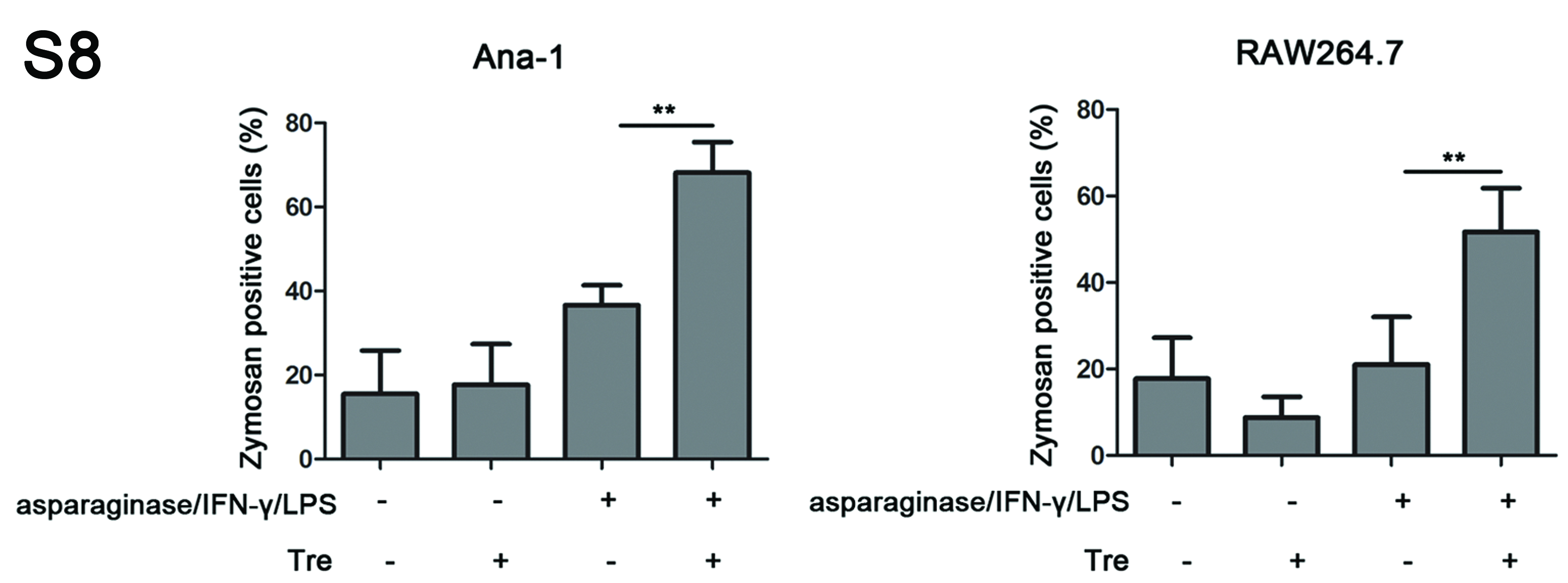


**Supplementary Figure 8. Activating autophagy overcomes asparaginase-induced immune suppression in** **Ana-1 and RAW264.7 cells.** Ana-1 and RAW264.7 cells were treated with 300 IU/mL IFN-γ, 200 ng/mL LPS and 0.1 IU/mL asparaginase, either alone or in combination with 25 µM Tre for 24 h. The cells were incubated with zymosan particles for another 2 h, and analyzed by confocal fluorescent microscopy. The percentage of zymosan positive macrophages was presented in bar charts. Results were represented as mean ± SD (***P* < 0.01).


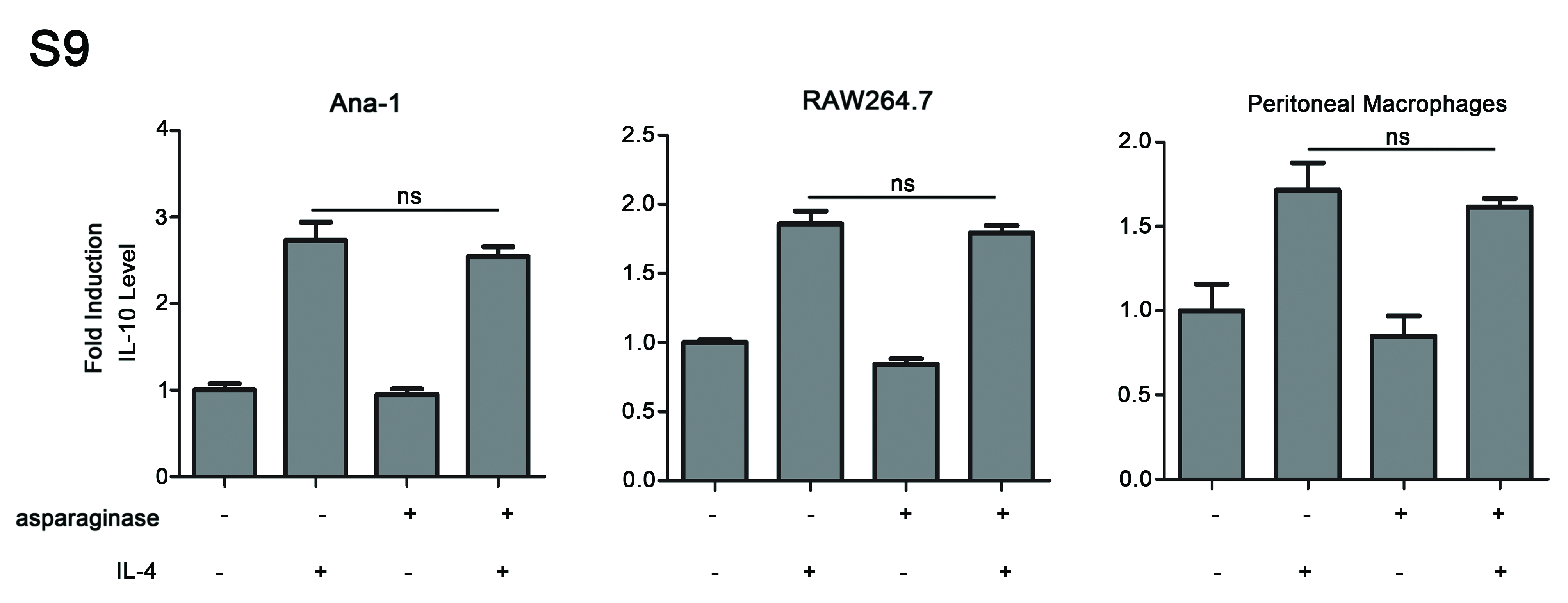


**Supplementary Figure 9. Treated with asparaginase has no effect on the cytokine secretion in macrophages.** Ana-1, RAW264.7 and [peritoneal](D:/%E8%BD%AF%E4%BB%B6/Dict/6.3.69.8341/resultui/frame/javascript:void(0);) [macrophage](D:/%E8%BD%AF%E4%BB%B6/Dict/6.3.69.8341/resultui/frame/javascript:void(0);)s were treated with 20 ng/mL IL-4, either alone or in combination with 0.1 IU/mL asparaginase for 24 h. The content of IL-10 in the supernatants of [macrophage](D:/%E8%BD%AF%E4%BB%B6/Dict/6.3.69.8341/resultui/frame/javascript:void(0);)s were measured by ELISA. Results were represented as mean ± SD.


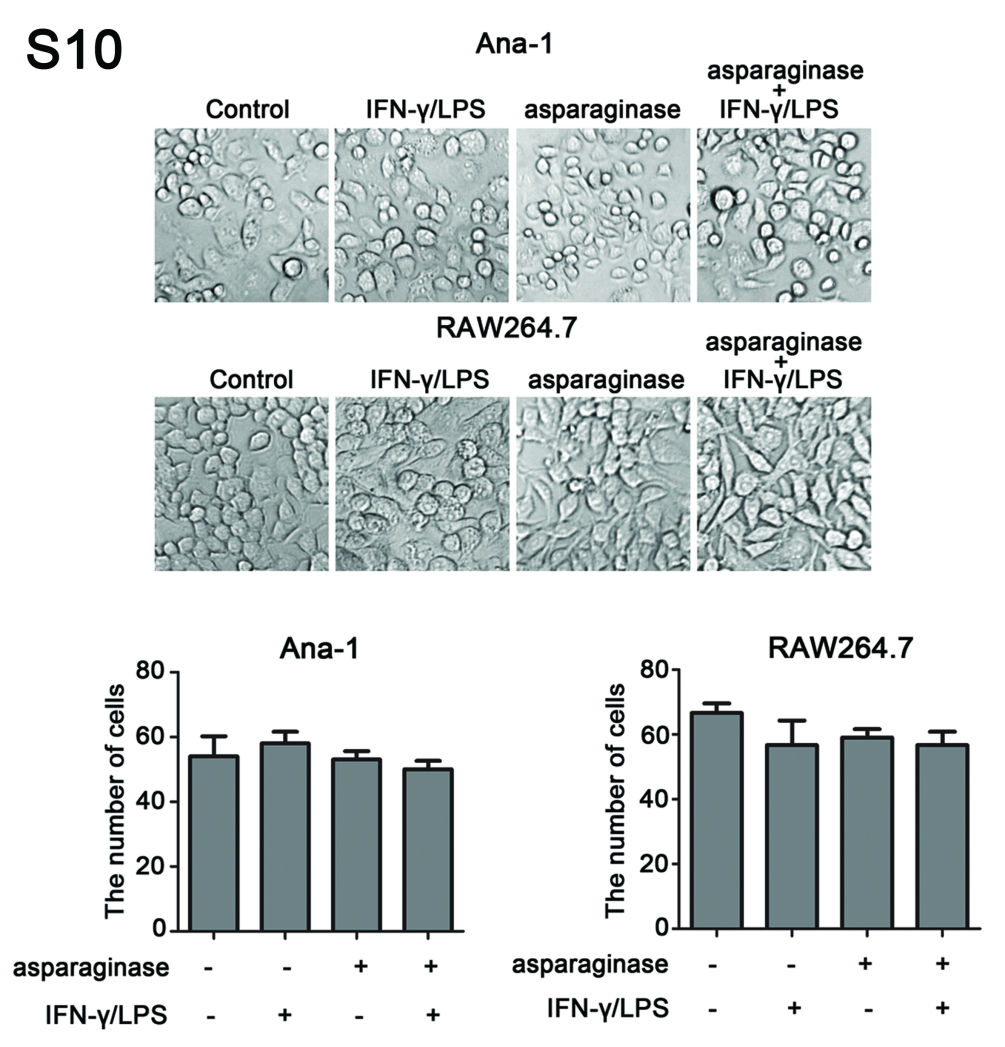


**Supplementary Figure 10. Treated with asparaginase has no effect on the number of Ana-1 and RAW264.7 cells.** Ana-1 and RAW264.7 cells were treated with 300 IU/mL IFN-γ and 200 ng/mL LPS, either alone or in combination with 0.1 IU/mL asparaginase for 24 h. Morphological and numerary changes of Ana-1 and RAW264.7 cells were observed using microscopy and photography. The number of normal cells was presented in bar charts.
